# Adherence to the Mediterranean diet and its protective effects against colorectal cancer: a meta-analysis of 26 studies with 2,217,404 participants

**DOI:** 10.1007/s11357-024-01296-9

**Published:** 2024-08-01

**Authors:** Zoltan Ungvari, Mónika Fekete, János Tibor Fekete, Giuseppe Grosso, Anna Ungvari, Balázs Győrffy

**Affiliations:** 1 Vascular Cognitive Impairment, Neurodegeneration and Healthy Brain Aging Program, Department of Neurosurgery, University of Oklahoma Health Sciences Center, Oklahoma City, OK USA; 2 Stephenson Cancer Center, University of Oklahoma, Oklahoma City, OK USA; 3 Oklahoma Center for Geroscience and Healthy Brain Aging, University of Oklahoma Health Sciences Center, Oklahoma City, OK USA; 4 Department of Health Promotion Sciences, College of Public Health, University of Oklahoma Health Sciences Center, Oklahoma City, OK USA; 5 International Training Program in Geroscience, Doctoral College/Institute of Preventive Medicine and Public Health, Semmelweis University, Budapest, Hungary; 6 Institute of Preventive Medicine and Public Health, Semmelweis University, Semmelweis University, Budapest, Hungary; 7 Department of Bioinformatics, Semmelweis University, 1094 Budapest, Hungary; 8 Cancer Biomarker Research Group, Institute of Molecular Life Sciences, HUN-REN Research Centre for Natural Sciences, H-1117 Budapest, Hungary; 9 Department of Biomedical and Biotechnological Sciences, University of Catania, Catania, Italy; 10 Center for Human Nutrition and Mediterranean Foods (NUTREA), University of Catania, Catania, Italy; 11 Department of Biophysics, Medical School, University of Pecs, H-7624 Pecs, Hungary

**Keywords:** Healthy aging, Dietary intervention, Malignant disease, Malignancies, Tumor, Unhealthy aging

## Abstract

Colorectal cancer (CRC) is a major global health concern and represents a significant public health challenge in Hungary, where it exhibits some of the highest morbidity and mortality rates in the European Union. The Mediterranean diet has been suggested to reduce the incidence of CRC, but comprehensive evidence from diverse study designs is needed to substantiate this effect. A systematic literature search was conducted in PubMed, ClinicalTrials.gov, CENTRAL, and the Web of Science to identify randomized controlled trials and human clinical trials from 2008 to 2024 to identify relevant studies. Statistical analysis was performed using the https://metaanalysisonline.com web application using a random effects model to estimate the pooled hazard rates (HRs). Forest plots, funnel plots, and *Z*-score plots were utilized to visualize results. We identified 15 clinical trials and 9 case–control studies, encompassing a total of 2,217,404 subjects. The pooled analysis indicated that adherence to the Mediterranean diet significantly reduced the prevalence of CRC (HR = 0.84, 95% CI = 0.78–0.91, *p* < 0.01). This protective effect was consistent across sexes, with HRs of 0.85 (95% CI = 0.75–0.97, *p* = 0.01) for males and 0.88 (95% CI = 0.79–0.99, *p* = 0.03) for females. Case–control studies specifically showed a substantial effect (HR = 0.51, 95% CI = 0.38–0.68, *p* < 0.01). Notable heterogeneity was observed across studies, yet the a priori information size was substantially below the cumulative sample size, ensuring sufficient data for reliable conclusions. The findings from this meta-analysis reinforce the protective role of the Mediterranean diet against CRC. The results of this meta-analysis will inform dietary interventions designed to mitigate CRC risk, which are conducted within the framework of the Semmelweis Study, an ongoing comprehensive cohort study at Semmelweis University, designed to explore the multifaceted causes of unhealthy aging in Hungary. These interventions aim to explore the practical application of Mediterranean dietary patterns in reducing CRC incidence among the Hungarian population.

## Introduction

Colorectal cancer (CRC) remains one of the most prevalent and lethal malignancies worldwide, with rising incidence rates particularly marked in the European Union (EU) [1–4]. CRC is increasingly recognized as an age-related disease, with the majority of cases diagnosed in individuals over the age of 50 [2, 4, 5]. This correlation underscores the importance of fundamental cellular and molecular aging processes in the development of CRC, where DNA damage and epigenetic dysregulation become prevalent over time, facilitated by chronic inflammation and oxidative stress [5, 6].

In Hungary, the unfavorable epidemiological situation—characterized by some of the highest rates of CRC in the European Union—can largely be attributed to the unhealthy aging of its population [7, 8]. A critical factor contributing to this phenomenon is lifestyle, particularly dietary habits [8–17]. The typical Hungarian diet, high in processed foods and low in fresh fruits and vegetables, aligns poorly with the principles of healthy aging [12, 13]. This dietary pattern not only exacerbates the risk factors associated with aging but also directly influences the incidence of age-related diseases like CRC [4, 18, 19]. Thus, the prevailing health challenges in Hungary, particularly the high prevalence of CRC, are a stark indicator of the broader issues stemming from an unhealthy lifestyle, with poor diet playing a pivotal role. This disturbing trend underscores the urgent need for effective preventive strategies and interventions tailored to the Hungarian population.

The Mediterranean diet is celebrated for its health benefits, primarily due to its rich composition of fruits, vegetables, whole grains, and healthy fats, primarily olive oil, along with moderate consumption of fish and poultry [20]. Numerous epidemiological studies have linked adherence to the Mediterranean diet with reduced incidences of chronic diseases, notably cardiovascular diseases and various forms of cancer, including colorectal carcinoma [16, 21–28]. The protective effects of the Mediterranean diet are often attributed to its high content of dietary fiber, antioxidants, and anti-inflammatory compounds, which are thought to play crucial roles in cancer prevention. In spite of its benefits, current evidence shows a progressive abandonment of this dietary pattern in favor of more “Westernized” dietary habits, representing a potential threat to human health and the local environment [29, 30].

In response to the critical health challenge posed by unhealthy aging in Hungary, the Semmelweis Study was initiated by Semmelweis University [8]. This prospective epidemiological survey aims to identify the key determinants of unhealthy aging in Hungary, with a particular focus on dietary patterns. Given the promising protective effects of the Mediterranean diet against CRC demonstrated in other populations [16, 31–55], there is a compelling rationale to explore whether these benefits can be replicated within the Hungarian context. Thus, this meta-analysis seeks to synthesize the available data on the association between the Mediterranean diet and CRC risk reduction, providing a solid scientific foundation to inform the design and implementation of dietary interventions under the Semmelweis Study framework. The ultimate goal is to leverage the insights gained to promote the Mediterranean diet as a viable strategy to address the unfavorable epidemiological trends observed in Hungary, potentially paving the way for significant public health improvements.

## Methods

### Literature screening

**Fig. 1:**
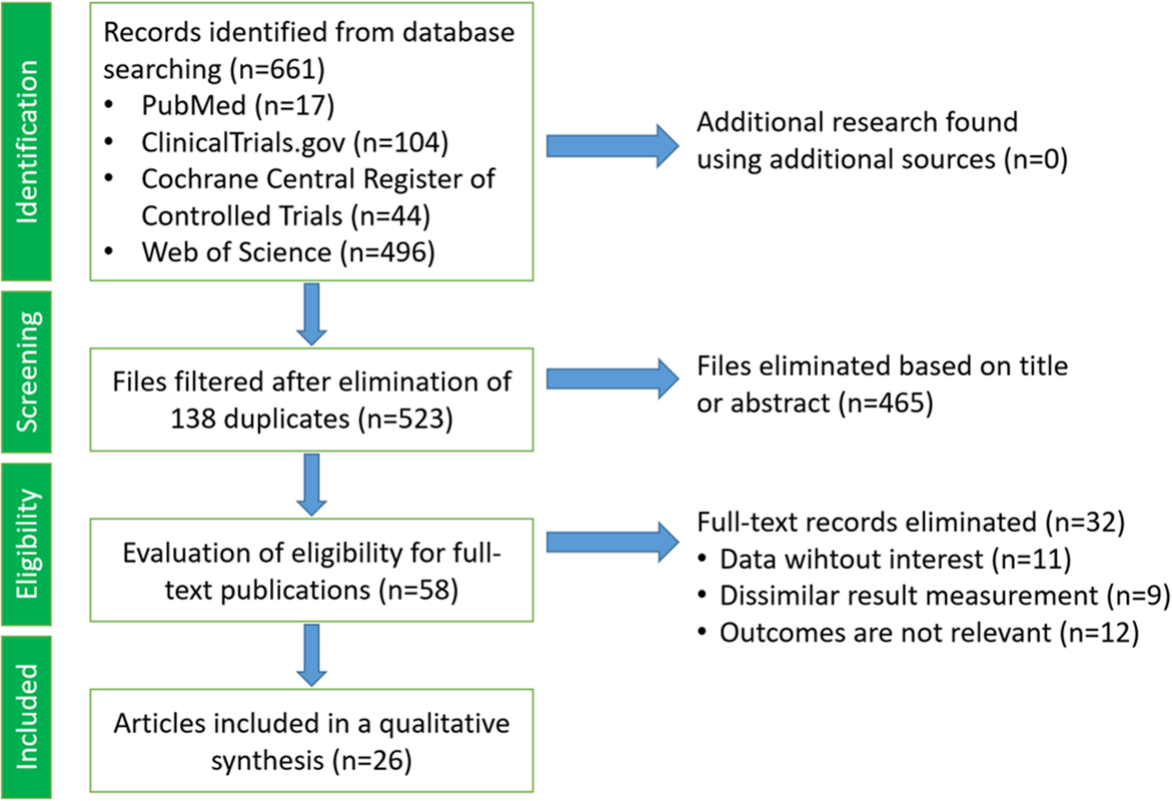
Flow diagram illustrating the article selection process

A systematic literature search was conducted in PubMed, ClinicalTrials.gov, the Cochrane Central Register of Controlled Trials (CENTRAL), and the Web of Science databases to identify randomized controlled trials (RCTs) and human clinical trials from 2008 to 2024. The search utilized specialized and MESH (Medical Subject Headings) keywords, including “Mediterranean diet,” “Mediterranean,” “colorectal,” “rectal,” “colon,” “tumor,” “carcinoma,” “cancer,” “neoplasm,” and “risk.” Language restrictions were not imposed. The search terms were combined using the conjunctions “AND” or “OR.” Duplicate articles were identified and removed based on indexing, following which titles and abstracts were screened for relevance. Articles not meeting the inclusion criteria, including those related to cancer mortality, benign adenoma, adenoma recurrence, benign polyp, and cancer survivor follow-up, were excluded. Selected articles underwent a thorough evaluation based on their full texts. The study selection process is depicted in Fig. 1.

### Determining the overall effect

Statistical analysis was performed using the https://metaanalysisonline.com web application. The random effects model was employed to estimate the pooled hazard rates (HRs) and odds ratios (ORs) and their 95% confidence intervals (CIs). Forest plots were drawn to visualize the individual studies as well as the summary results. Heterogeneity of the included studies was assessed by the chi-square test and *I*2 index. The forest plot concisely presents data from separate studies, offering a graphical depiction of the variability within the research and showcasing the projected overall effect.

A funnel plot was utilized to explore the correlation between the estimated effects from each study and the precision of those estimates. The funnel plots were drawn to analyze publication bias. Egger’s test was computed to estimate significance.

### Determining sample size robustness

We also performed a trial sequential analysis (TSA) for the a priori information size (APIS) under a risk ratio reduction of 15% with two-sided α = 5% and 1 – β = 80% power [56]. TSA analyses were done in Stata 14.1 using the metacoumbounds package. A *Z*-score plot was drawn to visualize the correlation between the aggregate sample size, time, and the cumulative *Z*-scores. The TSA analysis was performed for assessing whether the cumulative sample size is adequate to draw a conclusive inference or if further studies are required.

### Subcohort analysis settings

Each step of the entire statistical analysis was performed in four settings: a combined analysis was made using the aggregate of all studies, an analysis was made using only results obtained in men and women, and finally, a separate analysis was made for case–control studies—e.g., where individuals with a CRC (cases) are compared with individuals without the CRC (controls).

## Results

### Combined analysis

Altogether, 26 studies were identified, of which 15 studies were synthesized in the combined setting. Based on the analysis performed using the random effects model with inverse variance method to compare the HR, there is a statistical difference, and the summarized HR is 0.84 with a 95% confidence interval of 0.78–0.91. The test for overall effect shows a significance below 0.01. There is a significant heterogeneity among the study results with an *I*^*2*^ value of 76%. Overall, this indicates that a Mediterranean-style diet can reduce colorectal cancer incidence by 16%.

The funnel plot for the combined analysis indicates a potential publication bias. The Eggers’ test supports the presence of funnel plot asymmetry (intercept − 2.06, 95% CI − 3.6 to − 0.53, *t* − 2.644, *p*-value 0.02). Notably, smaller studies seem to overestimate the protective effects of the Mediterranean diet.

With a relative risk reduction of 15% and an alpha of 5%, the *Z*-score plot indicated that the APIS was at 119,390 samples. The actual size of the combined cohorts includes 2,196,631 samples. This indicates that the combined dataset is highly robust and further studies are unlikely to change the study results.

**Fig. 2:**
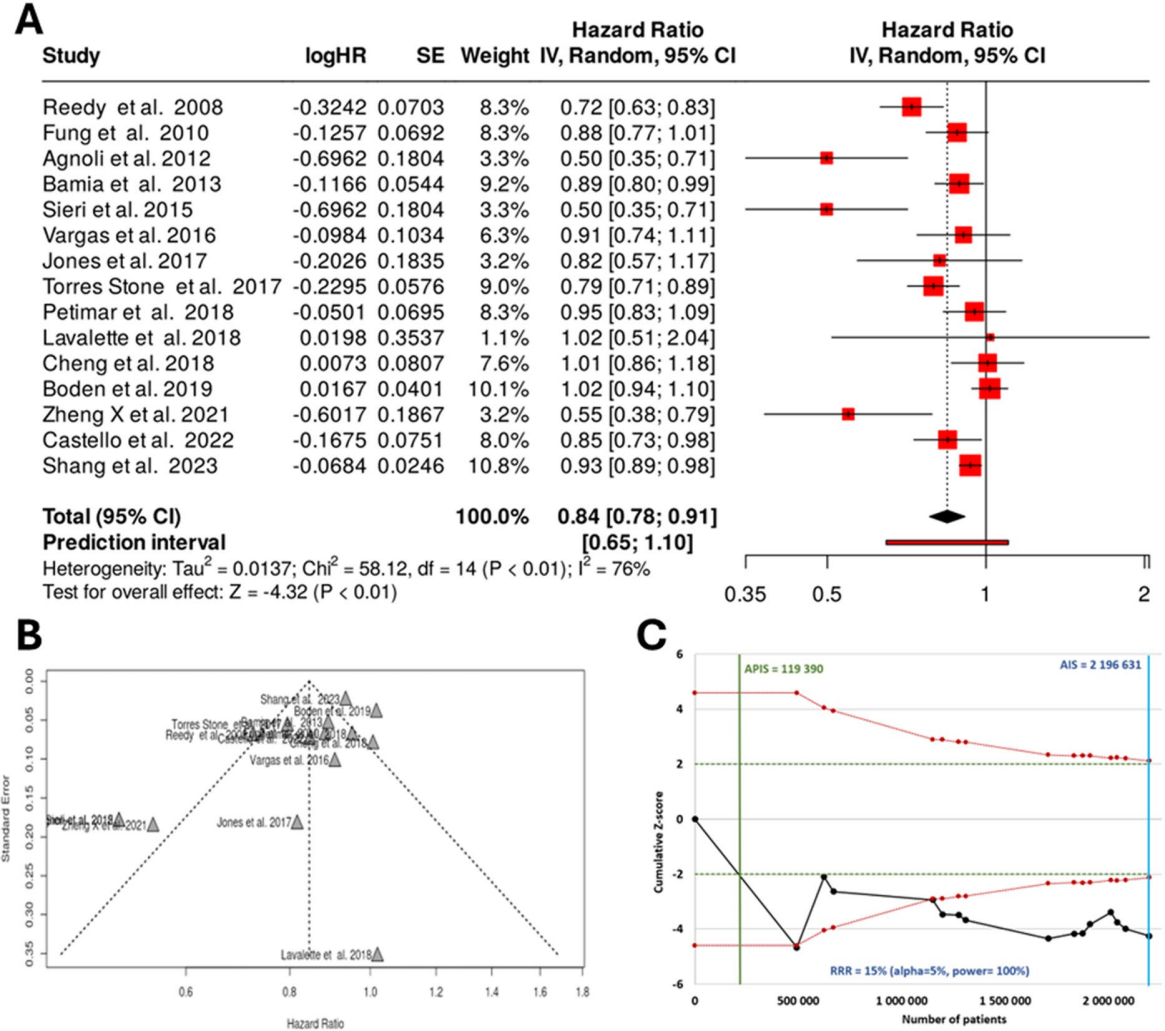
Meta-analysis results of assessing the correlation between a Mediterranean-style diet and colorectal cancer prevalence for the combined cohort for all available studies including a forest plot (**A**), a funnel plot (**B**), and a *Z*-score plot (**C**). SE, standard error; CI, confidence interval; IV, inverse variance; HR, hazard rate; APIS, a priori information size; AIS, actual information size; RRR, relative risk ratio

Each of the plots for the combined analysis is provided in Fig. 2.

### Male and female cohort analysis

**Fig. 3:**
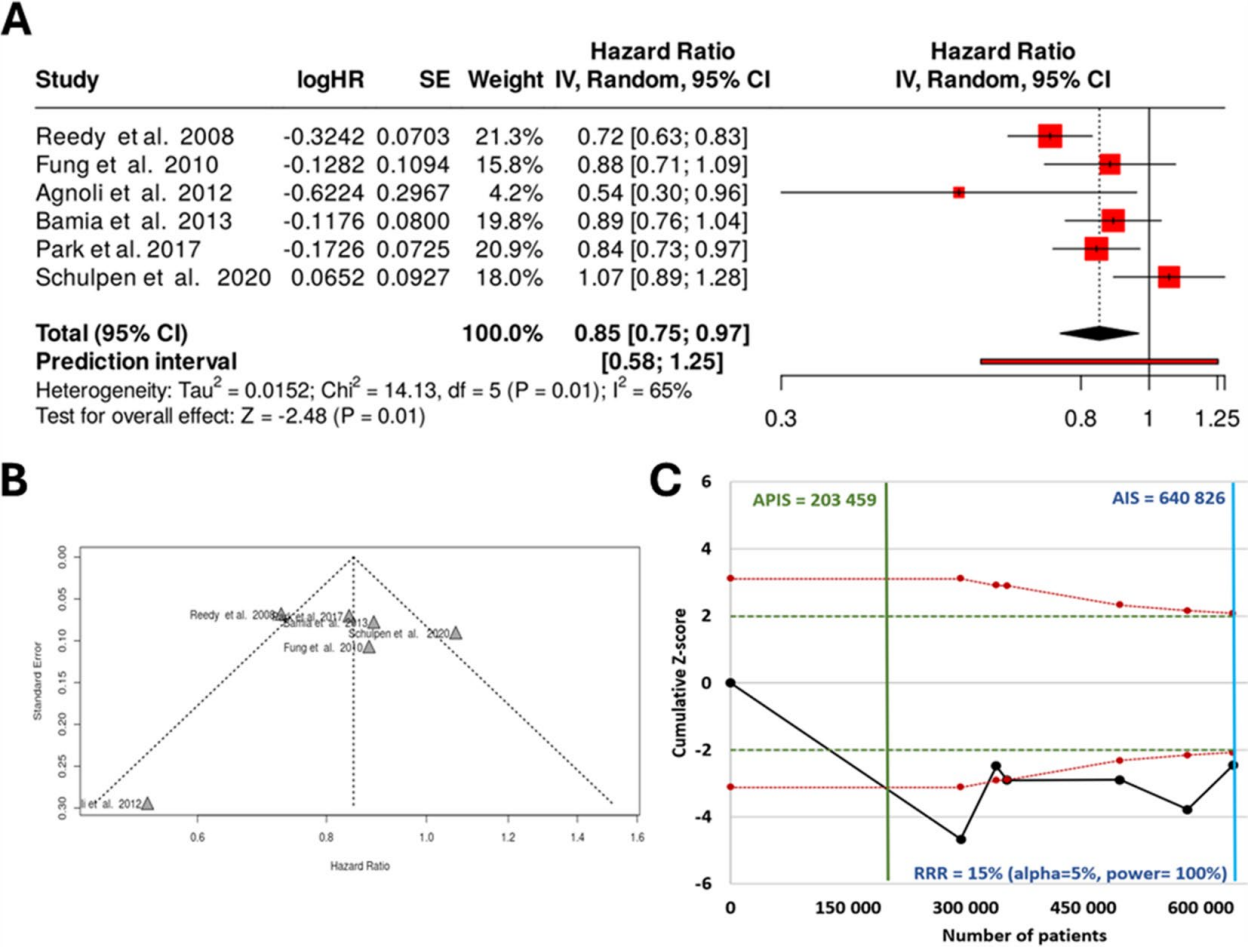
Meta-analysis results assessing the correlation between a Mediterranean-style diet and colorectal cancer prevalence in males only for the combined cohort for all available studies including a forest plot (**A**), a funnel plot (**B**), and a *Z*-score plot (**C**). SE, standard error; CI, confidence interval; IV, inverse variance; HR, hazard rate; APIS, a priori information size; AIS, actual information size; RRR, relative risk ratio

We next performed the analysis separately for the available male and female cohorts. In the male cohort, all together six studies were analyzed. Utilizing a random-effects model with the inverse variance method to compare the hazard rates, we identified a statistically significant difference. The summarized hazard rate was 0.85 with a 95% confidence interval ranging from 0.75 to 0.97, and the test for overall effect was significant (*p* = 0.01). Examination of the funnel plot for these male cohorts suggested no potential publication bias, corroborated by Eggers’ test which did not indicate funnel plot asymmetry (intercept − 0.57, 95% CI − 5.14 to 3.99, *t* − 0.246, *p*-value 0.818). The *Z*-score plot yielded an APIS of 203,459, significantly lower than the threshold of 640,826, indicating that the data from these male cohorts is sufficient for robust conclusions. Detailed results for the male cohort analysis are presented in Fig. 3.

**Fig. 4:**
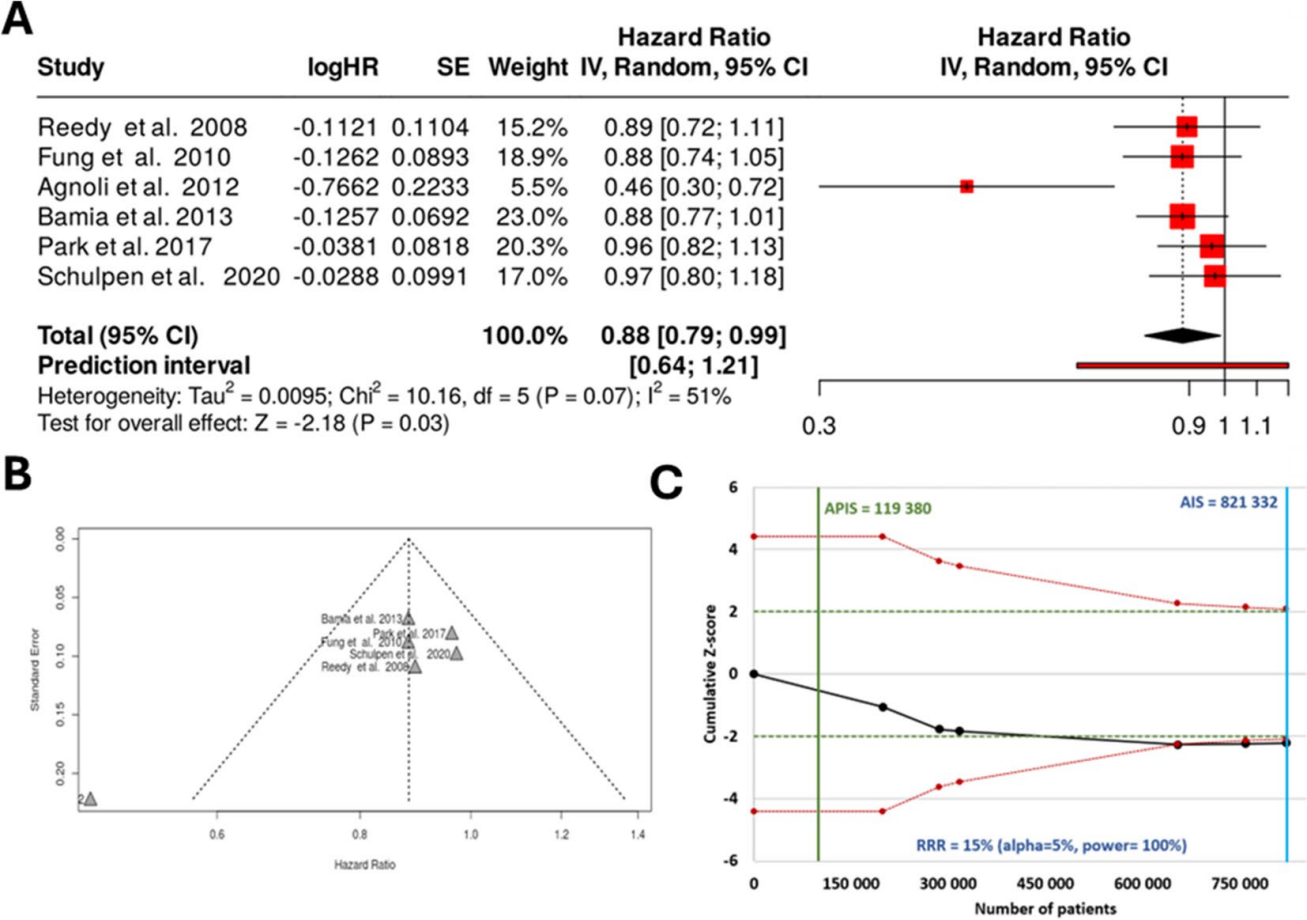
Meta-analysis results assessing the correlation between a Mediterranean-style diet and colorectal cancer prevalence in females only for the combined cohort for all available studies including a forest plot (**A**), a funnel plot (**B**), and a *Z*-score plot (**C**). SE, standard error; CI, confidence interval; IV, inverse variance; HR, hazard rate; APIS, a priori information size; AIS, actual information size; RRR, relative risk ratio

In the female cohort, a total of six studies were analyzed. Based on the analysis performed using the random effects model with inverse variance method to compare the hazard rate, there is a statistical difference, and the summarized hazard rate is 0.88 with a 95% confidence interval of 0.79–0.99. The test for overall effect is significant with *p* = 0.03. The funnel plot does not indicate a potential publication bias. The Eggers’ test does not support the presence of funnel plot asymmetry (intercept − 3.41, 95% CI − 6.31 to − 0.51, *t* − 2.302, *p*-value 0.083). The planned statistical threshold as presented in a *Z*-score plot had an APIS of 119,380 which is less than a quarter of the real value of 831,332 indicating a sufficient number of cases for concluding. The analysis results using all data gathered from females are provided in Fig. 4.

### Case–control

In the last analysis, we compared those studies where individuals with a CRC were compared to individuals without CRC (case–control studies). In this setting, all together nine studies were analyzed. Based on the analysis performed using a random effects model with inverse variance method to compare the hazard rate, there was a statistical difference, and the summarized hazard rate was 0.51 with a 95% confidence interval of 0.38–0.68. The test for overall effect delivered a significance with a *p* < 0.01. There is a substantial heterogeneity among the study results with an *I*^2^ value of 89%. The funnel plot indicates a potential publication bias. The Eggers’ test supports the presence of funnel plot asymmetry (intercept − 3.12, 95% CI − 4.43 to − 1.8, *t* − 4.64, *p*-value 0.002).

**Fig. 5:**
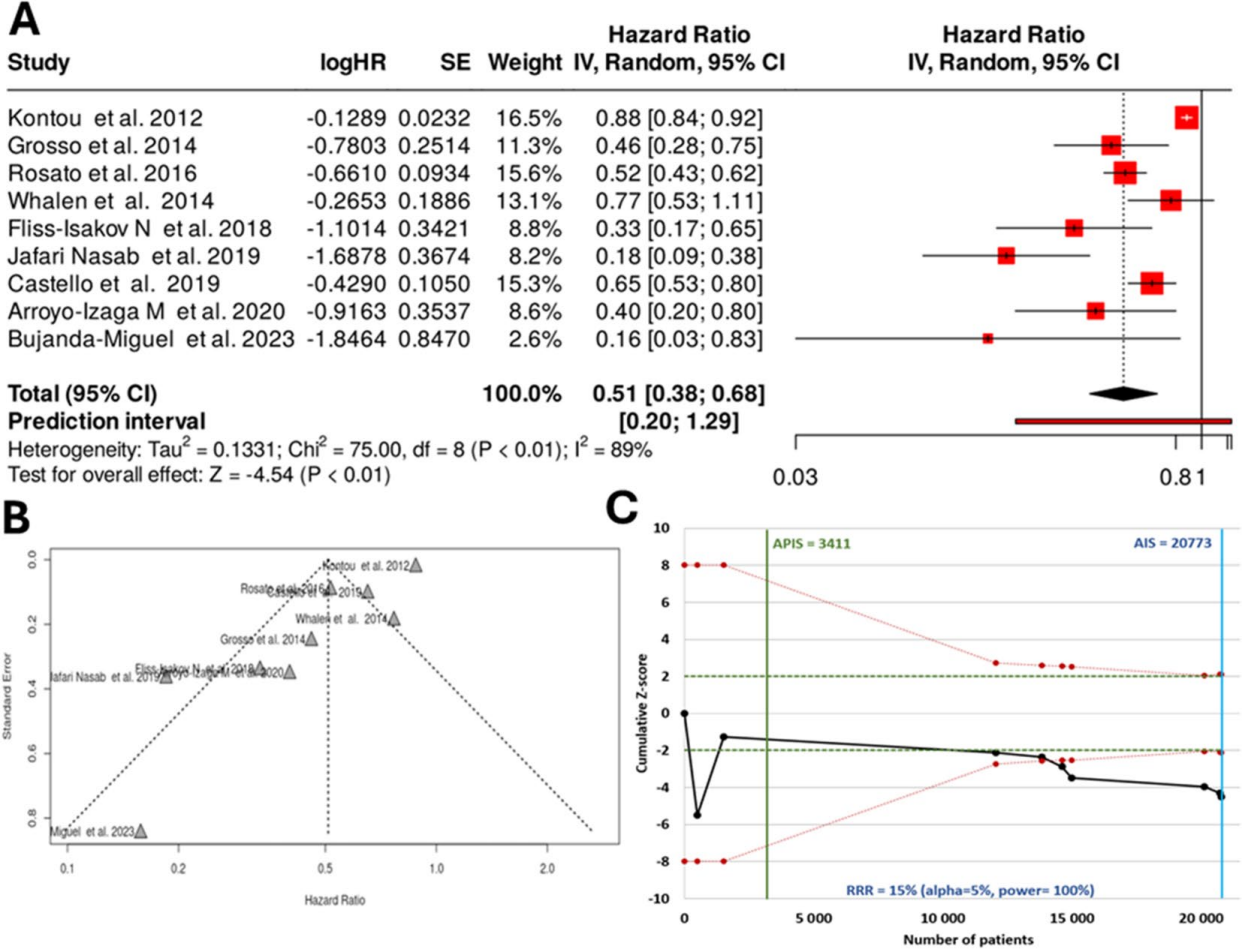
Meta-analysis results assessing case–control studies for evaluating the correlation between a Mediterranean-style diet and colorectal cancer with all available studies including a forest plot (**A**), a funnel plot (**B**), and a *Z*-score plot (**C**). SE, standard error; CI, confidence interval; IV, inverse variance; HR, hazard rate; APIS, a priori information size; AIS, actual information size; RRR, relative risk ratio

The intended statistical threshold depicted in a *Z*-score plot shows an APIS of 3411, substantially below the actual value of 20,773, suggesting an ample number of cases for conclusive inference. Figure 5 illustrates the analysis outcomes utilizing all data collected from the case–control studies.

## Discussion

This meta-analysis has demonstrated that high adherence to the Mediterranean diet is associated with a significant reduction in the risk of colorectal cancer, confirming the role of the Mediterranean diet as a key preventative strategy against this disease. The pooled relative risk suggests a 15 to 49% reduction in CRC risk for individuals adhering closely to Mediterranean dietary patterns. These results align with existing literature [16, 31–55], underscoring the anti-inflammatory and antioxidative properties of the Mediterranean diet, which likely contribute to its protective effects against cancer formation. For example, a similar protective effect was observed in the European Prospective Investigation into Cancer and Nutrition (EPIC) study, which highlighted dietary fibers and polyphenols in the Mediterranean diet as significant protective factors against CRC [39, 50]. The findings are consistent with previous studies that have identified the Mediterranean diet as beneficial in reducing the incidence of various chronic diseases, including other types of cancers and cardiovascular diseases [23–28, 57, 58].

The Mediterranean diet is thought to confer protection against cancer through several cellular and molecular mechanisms that modulate the aging process. Key components of the Mediterranean diet, such as polyphenols [59] and fibers [60], play pivotal roles in these protective mechanisms. Polyphenols, abundant in olive oil [61–65], nuts, and red wine, are known for their anti-inflammatory and antioxidant properties. Many of them activate sirtuins [66–68], including SIRT1, a protein that has been linked to longevity and epigenetic rejuvenation. SIRT1 is a histone deacetylase that influences DNA repair, mitochondrial function, inflammatory pathways, and gene expression related to aging and cancer, potentially delaying the onset of age-related diseases including CRC [69, 70].

Furthermore, the anti-inflammatory effects of the Mediterranean diet are crucial, as chronic inflammation is a known risk factor for CRC [14, 15, 41, 71]. In addition to the anti-inflammatory effects elicited by polyphenols, the high fiber content of the Mediterranean diet reduces pro-inflammatory markers and insulin resistance [72–74], while enhancing gut microbiota diversity [75], which in turn reduces the gut inflammation that can lead to cancerous mutations. Omega-3 fatty acids [76–78], predominantly from fish in the Mediterranean diet, may also contribute to reduced inflammation and inhibit the pro-inflammatory pathways involved in cancer progression.

Mitochondrial dysregulation and dysfunction are pivotal in aging and the development of age-related diseases [79–82]. As powerhouses of the cell, mitochondria are crucial for energy production, and their dysfunction leads to increased oxidative stress and impaired cellular metabolism, which contribute to the pathogenesis of diseases such as cancer [79, 83–85]. Components of the Mediterranean diet support mitochondrial health, including coenzyme Q10, polyphenols, and omega-3 fatty acids, which help reduce mitochondrial oxidative stress and mitochondrial DNA damage, maintain the integrity of the mitochondrial membrane, and enhance energy production efficiency [86]. Healthy mitochondria are essential for preventing oxidative stress and maintaining energy balance in cells, reducing the risk of mutations and the onset of cancer [79, 83–85].

Another significant mechanism through which the Mediterranean diet protects against CRC involves the reduction of oxidative stress, through the activation of the nuclear factor erythroid 2-related factor 2 (Nrf2) pathway [87–89]. Nrf2 is a transcription factor that regulates the expression of antioxidant proteins and ROS detoxifying enzymes, thereby protecting cells from oxidative macromolecular damage caused by free radicals [90–98]. Components of the Mediterranean diet can induce mild increases in ROS production, which triggers a Nrf2-mediated hormetic response that strengthens cellular defenses against more severe oxidative stress [88, 99]. This process, facilitated by phytochemicals like resveratrol from grapes and berries, enhances cellular resilience and longevity [88, 100, 101]. The resulting reduction in oxidative stress is essential for preventing oxidative damage to DNA, proteins, and lipids, which can accumulate over time and contribute to both aging and oncogenesis. Oxidative DNA damage is crucial for the induction of cellular senescence [102], and aging is associated with an increased burden of senescent cells, which support cancer development through the secretion of pro-inflammatory and pro-tumorigenic factors known as the senescence-associated secretory phenotype (SASP) [6, 103–108].

Autophagy is a cellular process that recycles damaged cellular components to generate energy [109, 110]. In the early stages of tumor development, autophagy acts primarily as a tumor suppressor. It helps maintain cellular homeostasis and integrity by removing damaged organelles, such as dysfunctional mitochondria, thus preventing increased mitochondrial production of ROS that are mutagenic. The Mediterranean diet, particularly when combined with time-restricted eating or intermittent fasting, may enhance autophagy, facilitating the removal of dysfunctional proteins and organelles [111–113].

The mechanistic Target Of Rapamycin (mTOR) pathway is crucial for cell growth and proliferation [114, 115]. Overactivation of this pathway is linked to aging and cancer [114]. The Mediterranean diet, through its balanced protein content and high levels of polyunsaturated fats and phytochemicals, may downregulate mTOR signaling [116], thus inhibiting cell proliferation and survival of cancerous cells.

Additional mechanisms by which the Mediterranean diet may protect against tumorigenesis include its beneficial effects on the gut microbiota, which play a crucial role in colon health and overall disease prevention [117–120]. The high fiber content of the Mediterranean diet, sourced from a variety of fruits, vegetables, and whole grains, significantly alters the composition of the gut microbiota. This dietary fiber is fermented by gut bacteria, producing short-chain fatty acids (SCFAs) such as butyrate, propionate, and acetate. Among these, butyrate is particularly noteworthy for its role as a primary energy source for colonocytes and its potent anti-inflammatory and anti-carcinogenic properties [121, 122].

Components of the Mediterranean diet may also play a role in suppressing the growth and virulence of oncogenic bacteria, such as *Fusobacterium nucleatum*, which are implicated in the development and progression of colorectal cancer [118, 119, 123–126]. *Fusobacterium* spp. are known to promote tumor growth by modulating the tumor microenvironment, inducing inflammation, and interacting with cancer cells to enhance their growth and invasiveness [126–132]. The impact of the Mediterranean diet on reducing these harmful bacterial populations while enhancing overall microbial diversity and stability is critical.

The sex-specific analysis within this study indicated that men benefit more from the Mediterranean diet in terms of CRC risk reduction compared to women. This could be due to biological differences, such as hormonal influences that modulate diet effectiveness, or behavioral differences in diet adherence [133–138]. Future research should focus on understanding these mechanisms and exploring whether dietary recommendations should be adjusted to maximize efficacy in each sex.

One of the strengths of this meta-analysis is its large sample size and the inclusion of studies from diverse geographical locations, enhancing the generalizability of the findings. Additionally, the robustness of the results was confirmed through rigorous sensitivity analyses, indicating that the observed associations are stable across various study designs and populations. However, the study is not without limitations. Despite adjusting for major confounders, residual confounding cannot be entirely ruled out. Dietary self-reporting, which is prone to measurement errors, was used in the original studies, potentially leading to misclassification of dietary adherence. The moderate heterogeneity observed in our meta-analysis indicates substantial variability in how the Mediterranean diet is defined and measured across different studies. This variability likely arises from a combination of factors, including diverse dietary assessment methods, regional variations in dietary practices that fall under the broad category of the Mediterranean diet, and differing baseline characteristics of the study populations, such as age, sex, and health status. These factors can significantly affect the results and interpretations of the diet’s impact on colorectal cancer risk. Understanding and addressing these sources of heterogeneity is crucial not only for accurately assessing the protective effects of the Mediterranean diet but also for refining dietary recommendations to ensure they are effectively tailored to specific demographic and regional contexts. This nuanced understanding will enhance the precision of public health strategies aimed at cancer prevention through dietary modifications.

The findings from this meta-analysis have significant implications for the Semmelweis Study, a comprehensive, prospective cohort study conducted at Semmelweis University [8]. The unhealthy aging of the Hungarian population is likely influenced by a complex interplay of factors, not all of which are fully understood. Behavioral risk factors such as low physical activity, obesity, and unhealthy diet are particularly prevalent in Hungary, accounting for half of all deaths, a rate significantly higher than the European Union average of 39% [139]. The Semmelweis Study aims to dissect the multifaceted influences of lifestyle factors on the unsuccessful aging process and the progression of chronic age-associated diseases within the Hungarian population [8]. A primary component of the Semmelweis Study’s baseline data collection includes a comprehensive questionnaire with a health determinants module that collects detailed data on dietary habits among participants [8]. This data collection is crucial as it directly connects with the substantial benefits observed from the Mediterranean diet in mitigating risk factors associated with aging and chronic diseases, such as CRC and cardiovascular disease. The findings of the present meta-analysis align closely with the study’s objectives to explore how dietary patterns influence health outcomes in the aging Hungarian population. Integrating detailed assessments of dietary adherence into the study’s initial and subsequent data collection phases will allow the investigative team to more effectively gauge the impact of diet on aging and disease outcomes. This methodological approach not only facilitates the identification of significant correlations but also supports the exploration of causal relationships through detailed longitudinal analysis. By doing so, the study can offer valuable insights into how dietary factors contribute to successful or unsuccessful aging, informing targeted interventions and public health strategies aimed at improving the well-being of the Hungarian population. Investigating how the Mediterranean diet’s impact varies between male and female employees could uncover important biological or behavioral factors that influence dietary effectiveness. Another intriguing aspect for exploration is the interplay between lifestyle factors such as diet and the occupational and environmental conditions encountered by the university’s employees. Understanding how these factors collectively impact aging and health outcomes is crucial. For instance, the study could examine whether certain occupational stressors or environmental exposures at the university amplify the effects of the diet or perhaps obscure its benefits. Additionally, understanding how the Mediterranean diet interacts with lifestyle variables like physical activity levels, smoking, and alcohol consumption could provide a holistic view of its role in promoting health and longevity in the workplace.

To further enhance our understanding, embedded studies within the Semmelweis Study are being meticulously designed to specifically explore the impact of the Mediterranean diet within the Hungarian context. These embedded studies aim to assess dietary adherence and examine specific components of the Mediterranean diet [140–144] such as the intake of olive oil, fruits, vegetables, nuts, and legumes. Additionally, pilot interventional studies are planned where subsets of participants are encouraged to adopt a Mediterranean diet. These trials will monitor changes in health indicators to longitudinally assess the diet’s efficacy. Studies will consider the socioeconomic and cultural factors that may facilitate or hinder the adoption of the Mediterranean diet among Hungarians. This aspect is crucial for tailoring dietary recommendations that are feasible and effective within the local context. By incorporating these targeted studies, the Semmelweis University initiative not only aligns with global health strategies but also pioneers a region-specific approach to combating age-related diseases through dietary interventions. This strategic focus on the Mediterranean diet could lead to significant public health benefits, providing a model for similar interventions in other populations facing comparable health challenges.

The enhanced focus on the Mediterranean diet within the Semmelweis Study has the potential to shape targeted dietary interventions that could substantially decrease the incidence of age-related diseases among the university’s employees. Moreover, by pinpointing the dietary factors that differentiate successful from unsuccessful aging, the study could yield vital insights that enhance public health strategies, not only for the university community but also for the broader population. The demonstration of the Mediterranean diet’s protective role against CRC carries profound implications for public health, especially in Hungary, where rates of CRC morbidity and mortality are alarmingly high. The adoption and promotion of the Mediterranean diet could become a cornerstone of national cancer prevention strategies. This dietary approach not only aligns with global health recommendations but also offers a culturally adaptable model for improving health outcomes through nutrition. Thus, the insights gained from the Semmelweis Study could inform broader dietary guidelines and public health policies aimed at combating one of the most prevalent cancers in Hungary.
